# Gas sensing properties of hematite nanoparticles synthesized *via* different techniques

**DOI:** 10.1039/d4ra02338b

**Published:** 2024-05-30

**Authors:** Mokhtar Hjiri, Saja Algessair, Ramzi Dhahri, Ali Mirzaei, Giovanni Neri

**Affiliations:** a Department of Physics, College of Sciences, Imam Mohammad Ibn Saud Islamic University (IMSIU) Riyadh 11623 Saudi Arabia mbhjiri@imamu.edu.sa m.hjiri@yahoo.fr +966506163909; b Department of Physics, Faculty of Sciences and Arts, Najran University P.O. Box 1988 Najran 11001 Saudi Arabia; c Department of Materials Science and Engineering, Shiraz University of Technology Shiraz Iran; d Department of Engineering, University of Messina Messina 98166 Italy

## Abstract

The synthesis techniques used for metal oxide semiconductors strongly influence their chemical, physical and gas sensing characteristics. In this context, hematite (α-Fe_2_O_3_) nanoparticles (NPs) were synthesized using two different techniques, namely, sol–gel (named H_SG_) and Pechini sol–gel (named H_PSG_). The average crystallite size and surface area were 15 nm and 76 m^2^ g^−1^ and 20 nm and 57 m^2^ g^−1^ for H_PSG_ and H_SG_, respectively. Morphological studies showed that the H_SG_ material was composed of ellipsoid-shaped particles, while the H_PSG_ material had peanut-shaped particles with open pores and channels. The comparison between the sensing performances of H_PSG_ and H_SG_ toward ethanol indicated H_PSG_ to be a better sensing material for ethanol detection. The H_PSG_ sensor exhibited a response of 12 toward 500 ppm ethanol at 250 °C, a fast response time of 5 s and excellent selectivity. The enhanced characteristics were mainly related to the peculiar morphology with a porous nature, which led to more gas adsorption and diffusion. In addition to shape influence, the size of NPs also has an effect on the gas sensing performance. In fact, a decrease in the crystallite size led to an increase in the surface area of the material where the gas molecule-sensing layer interaction took place. The increase in the surface area created more interaction sites, and thus the sensitivity was improved. From these results, the H_PSG_ sensor can be regarded as a promising candidate for ethanol detection.

## Abstract 2

The synthesis techniques used for metal oxide semiconductors strongly influence their chemical, physical and gas sensing characteristics.

## Graphical abstract

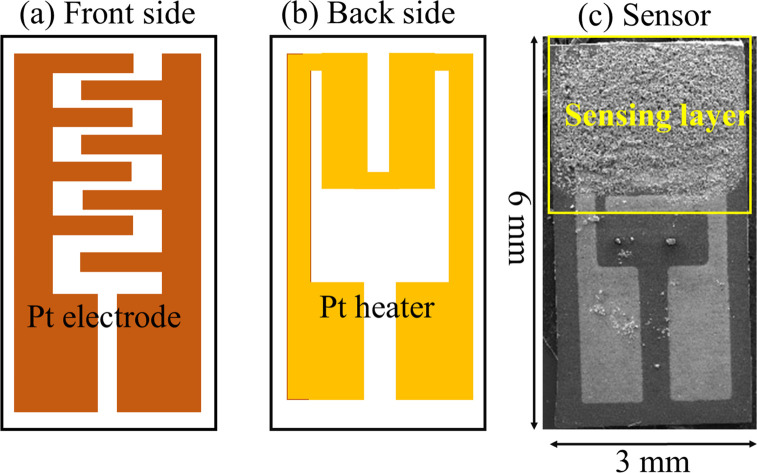

## 1 Introduction

Metal oxide semiconductors (MOSs) have been investigated for more than sixty years as gas sensing materials due to their resistance modulation upon the interaction between gas molecules and the adsorbed oxygen species on their surfaces.^1^ To date, numerous research studies have been performed on gas sensing applications of metal oxides such as ZnO,^2–4^ SnO_2_,^5–7^ NiO,^8,9^ WO_3_,^10,11^ TiO_2_,^12,13^ In_2_O_3_,^14,15^ and α-Fe_2_O_3_.^16^ Hematite (α-Fe_2_O_3_) is known as an n-type semiconductor with a 2.1 eV band gap.^17^ It is the most stable phase among iron oxide phases, with the properties of nontoxicity, abundance and low cost.^18^ All the above features make this material a promising candidate for gas sensing applications with enhanced performance.

To improve the sensing characteristics of hematite, numerous studies have been focused on preparing this material with different morphologies, sizes and shapes. The preparation techniques can influence the size, shape and morphology of nanomaterials and will eventually influence its sensing performance. Umar *et al.* synthesized α-Fe_2_O_3_ microstructures with a high response of 13 to 100 ppm ethanol at 400 °C, rapid response time (3 s) and high selectivity toward ethanol.^19^ Quasi-cubic hematite was prepared by Zhang and his colleagues *via* a one-pot solvothermal reaction and tested toward the detection of 100 ppm of ethanol at 285 °C. The fabricated sensor exhibited a good selectivity toward ethanol with a response of 17 and a response time of 12 s.^20^ In addition, Li and his team fabricated a selective H_2_S sensor based on hematite micro-ellipsoids synthesized using the hydrothermal technique.^21^ Besides, Hjiri *et al.* prepared hematite nanoparticles *via* the hydrothermal method and conducted sensing tests toward 5 ppm of NO_2_ gas; they found that the sensor was selective to NO_2_ with a high response of 60% at 200 °C and a fast response time of 10 s.^22^

In this context, hematite nanoparticles were synthesized *via* sol–gel and Pechini sol–gel routes and tested toward ethanol vapor monitoring. Ethanol (C_2_H_5_OH) is a volatile compound used in numerous industries and applications, including gasoline, solvents, detergents and disinfectants, and cleaning products. Ethanol is a hazardous compound to human life; for example, liver cancer risk is increased by high alcohol use and/or exposure, especially in women at high risk for breast cancer.^23^ Thus, there is an urgent demand for monitoring ethanol at low concentrations. The performances of the developed hematite sensors, in terms of response and selectivity, were compared to acquire information on the most promising sensor for monitoring ethanol, and the sensing mechanism was discussed as well.

## 2 Experimental details

### 2.1. Synthesis of α-Fe_2_O_3_ NPs *via* the sol–gel method (H_SG_)

The sol–gel procedure was adopted to produce iron oxide nanoparticles under supercritical ethanol conditions (*T*_c_ = 243 °C; *P*_c_ = 63.6 bar). Initially, 6 g of iron(iii) acetylacetonate (C_15_H_12_FeO_6_; MW = 353.17 g mol^−1^) was dissolved in 36 mL of methanol. Then, it was placed into an autoclave and dried under supercritical ethanol conditions after 15 min of stirring. Finally, crystalline hematite nanoparticles were produced by annealing at 550 °C for 3 h in air.

### 2.2. Synthesis of α-Fe_2_O_3_ NPs *via* the Pechini sol–gel method (H_PSG_)

The precursors and solvents used for hematite synthesis, purchased from Merck, were iron nitrate (Fe (NO_3_)_3_·9H_2_O; MW = 403.95 g mol^−1^), poly(vinyl pyrrolidone; MW = 40 000 g mol^−1^), citric acid (C_6_H_8_O_7_·H_2_O; CA; MW = 210.14 g mol^−1^) and ethylene glycol (C_2_H_6_O_2_; EG; MW = 62.07 g mol^−1^). Initially, distilled water was utilized to dissolve iron nitrate at 60 °C for 1 h. The solution was then combined with PVP solution in the molar ratio [PVP]/[Fe^3+^] = 1. Next, CA was dissolved in distilled water at 70 °C for 30 min. While stirring, the CA solution was gradually added to the PVP/Fe^3+^ solution. Then, with a molar ratio [citric acid]/[EG] = 2, EG was added to the above solution. The obtained solution was refluxed at 150 °C for 2 h. The precursor resin generated was dried at 120 °C for 12 h. Hematite was obtained after annealing at 550 °C for 3 h.

### 2.3. Characterization techniques

To assess the microstructural characteristics, the synthesized materials were characterized by X-ray diffraction (XRD; AXS D8 Advance; BRUKER, Billerica, MA, USA) using Cu Kα_1_ with a wavelength of 1.5405 Å. To obtain precise structural parameters, a Rietveld refinement analysis was conducted using the Match! Software. Morphological studies were performed using transmission electron microscopy (TEM, JEOL, JEOL 5600LV Tokyo, Japan) and scanning electron microscopy (SEM) experiments. Energy dispersive X-ray spectroscopy (EDS) was used to determine the composition of the samples.

### 2.4. Sensing tests

**Fig. 1:**
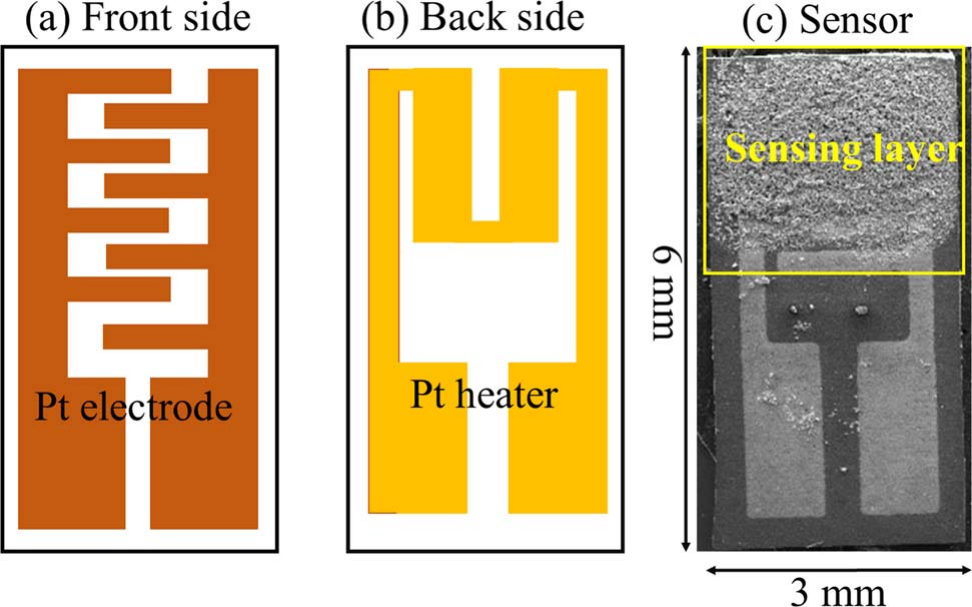
(a) Front side, (b) back side and (c) image of the fabricated sensor on the alumina substrate.

Alumina substrate (6 × 3 mm^2^) was used as a substrate; its front side was equipped with Pt interdigitated electrodes and in its back side, a Pt heater was used (Fig. 1). Thick films of hematite with a thickness of ∼10 μm were printed onto the Pt interdigitated electrodes.

To conduct sensing tests, the sensors were placed inside a test chamber made of stainless steel. The ethanol gas concentration was adjusted between 12.5 and 500 ppm using mass flow controllers. Under a total stream of 100 sccm, electrical measurements were made at 200 and 400 °C, gathering the sensor resistance data in the four-point mode. To this end, an Agilent 34970A multimeter data acquisition unit (Santa Clara, CA, USA) was utilized together with an Agilent E3632A dual-channel power supply instrument to bias the sensor's built-in heater and enable super-ambient temperature readings. Gas response was determined as *R* = *R*_a_/*R*_g_, where *R*_a_ denotes the baseline resistance in dry synthetic air, and *R*_g_ is the electrical resistance of the sensor in the presence of gas. The response time is the time required for the resistance to reach to its 90% final value in the presence of ethanol gas, and the recovery time is defined as the time needed for the resistance to come back to its 90% initial value after stoppage of ethanol gas.

## 3 Results and discussion

### 3.1. Microstructural and morphological characterizations

**Fig. 2:**
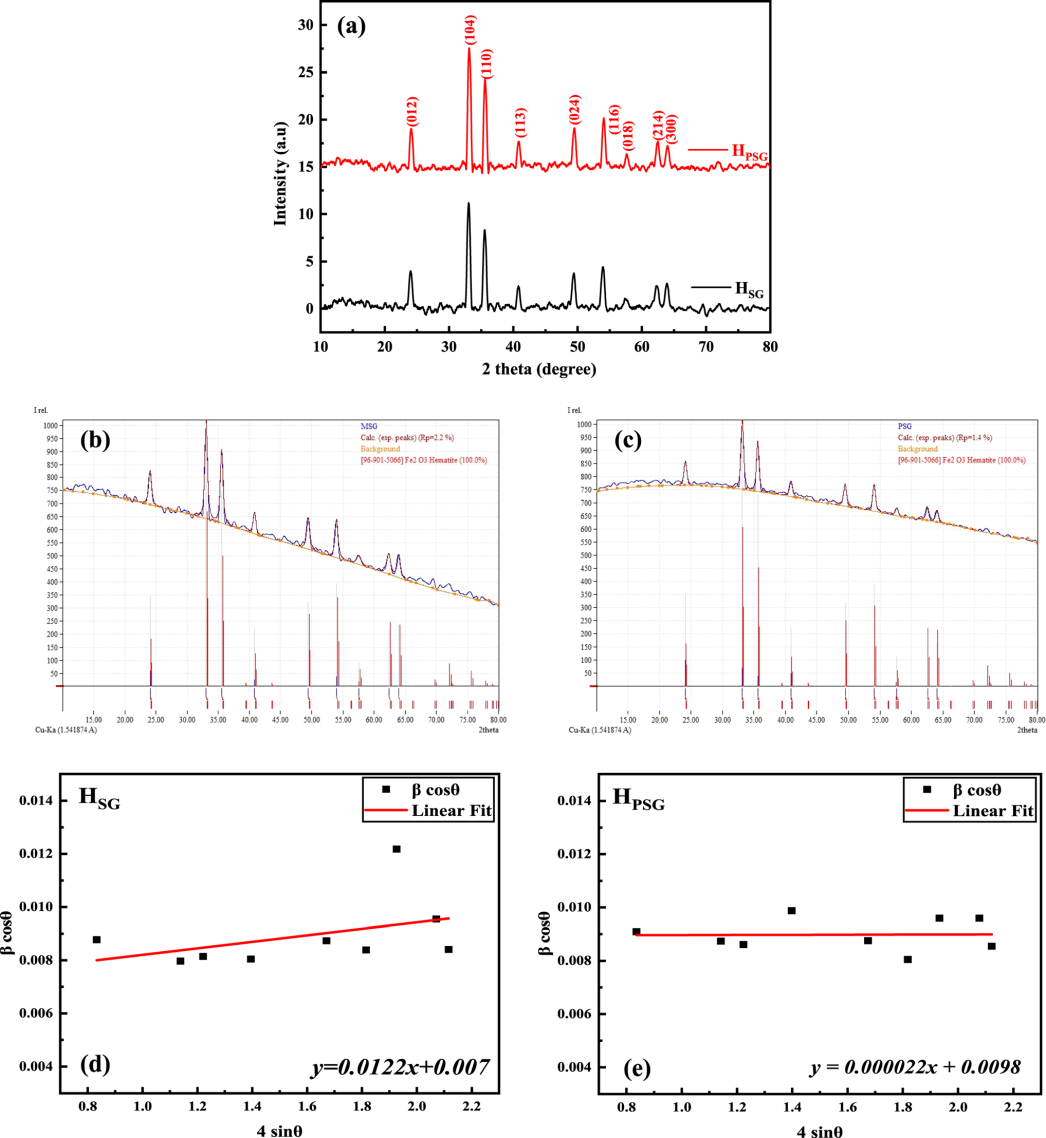
(a) XRD pattern of H_PSG_ and H_SG_ samples. (b) The Rietveld refinement plot of the H_PSG_ sample. (c) The Rietveld refinement plot of the H_SG_ sample (the blue curve represents the observed data and the oily color is the calculated pattern; the light blue curve shows the difference between the observed and calculated intensities). (d) Williamson–Hall plots of the H_PSG_ sample. (e) Williamson–Hall plots of the H_SG_ sample.

Fig. 2(a) displays the XRD patterns of the H_PSG_ and H_SG_ samples, revealing distinct diffraction peaks with varying intensities. These peaks are consistent with the trigonal structure of the phase of α-Fe_2_O_3_, according to JCPDS Card No. 96-901-5066. The corresponding 2θ peaks located at ∼24°, 33°, 35°, 41°, 50°, 54°, 58°, 62°, and 64° represent the crystal planes of (012), (104), (110), (113), (024), (116), (018), (214), and (300), respectively.^24,25^ No secondary phases were observed, indicating the high purity of the synthesized materials. As depicted in Fig. 2(b) and (c), Rietveld analysis was performed on the obtained XRD patterns. The refined lattice parameters and other relevant crystallographic data are summarized in Table 1.

**Table 1:** Crystallite sizes and structural parameters deduced from Rietveld analysis^a^

		Sample	
		H_PSG_	H_SG_
*D*_WH_ (nm)		15	20
Micro-strain (*ε*)		0.000022	0.00122
Space group		Trigonal	Trigonal
Crystal system		*R*3̄*c* (167)	*R*3̄*c* (167)
Density (g cm^−3^)		5.269	5.255
Volume, *V* (Å^3^)		301.9614	302.7998
Lattice parameters	*a* (Å)	5.0366	5.0383
	*c* (Å)	13.7452	13.7739

a There are no notable changes observed in the lattice parameters “*a*” and “*c*” between the H_PSG_ and H_SG_ samples.

The average crystallite size of both the samples was estimated using Williamson–Hall's method.^26^1
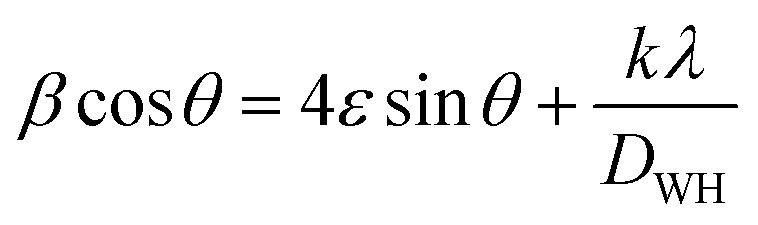


The average crystallite sizes as well as the microstrain values are provided in Table 1. Also, in Fig. 2(c) and (d), the Williamson–Hall plots of H_PSG_ and H_SG_ are presented. The H_SG_ sample exhibited larger crystallite sizes (20 nm) compared to those of the H_PSG_ sample (15 nm). This suggested that the operating conditions in the Pechini sol–gel technique can increase the nucleation centers for hematite particles' formation.

**Fig. 3:**
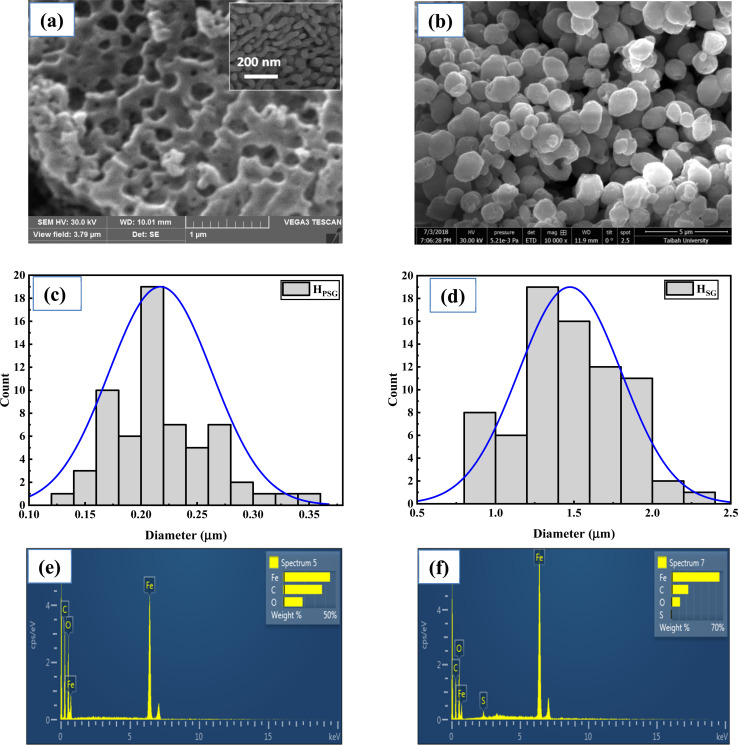
SEM images of the (a) H_PSG_ and (b) H_SG_ samples. Inset in (a) shows higher magnification image. Particles size distribution of the (c) H_PSG_ and (d) H_SG_ samples. EDS spectra of the (e) H_PSG_ and (f) H_SG_ samples.

Fig. 3(a) and (b) depicts the SEM images of the H_PSG_ and H_SG_ NPs, illustrating their distinct morphologies. The H_PSG_ sample exhibited many open spaces and channels; a higher magnification of the sample reveals the presence of small peanut-like NPs, while H_SG_ displayed granular ellipsoid-like particles. In fact, the voids between the Fe_2_O_3_ NPs prepared using the PSG method were formed during annealing at high temperature, where some particles agglomerated to form a larger porous particle. The average sizes of the hematite peanut and ellipsoid particles were 0.22 and 1.47 μm, respectively, as shown in Fig. 3(c) and(d). The difference between the shape and size of the Fe_2_O_3_ NPs obtained using two different synthesis methods is mainly related to the precursors used for the synthesis of H_PSG_ and H_SG_. For the samples prepared using the Pechini sol–gel method, CA as a chelating agent and EG as a polymer network former led to the isolation of Fe cations and small particle sizes, while the sample prepared using the sol–gel method without any chelating agent showed larger particle sizes. Also, the shapes of the synthesized samples are affected by the types of precursors used. However, further studies are necessary to shed light on the reasons for the final shapes of Fe_2_O_3_ NPs. The EDS spectra presented in Fig. 3(e) and (f) provide valuable insights into the elemental composition of the prepared materials. The Fe, O, and C elements appeared in both the samples, indicating the presence of the α-Fe_2_O_3_ phase.

Table 2 provides the amount of Fe, O, and C elements for both the samples obtained from EDS analysis. However, the carbon signal observed in the EDS spectra probably originated from the sample holder or from the environment. Also, EDS analysis for low atomic weight atoms such as C may have some errors. In this analysis, H_SG_ has higher concentrations of Fe and lower amount of O compared to H_PSG_. However, it should be noted that EDS is a point analysis and gives chemical composition in a selected point rather than an average chemical composition. The sulfur element in the H_SG_ sample was probably considered as a contaminant, coming from the autoclave container used for preparing several materials in our laboratory.

**Table 2:** Amount of different elements in the H_PSG_ and H_SG_ samples in weight percent

Sample	Fe	O	C	S
H_PSG_	45	19	36	—
H_SG_	65	10	22	3

### 3.2. Electrical and gas sensing studies

**Fig. 4:**
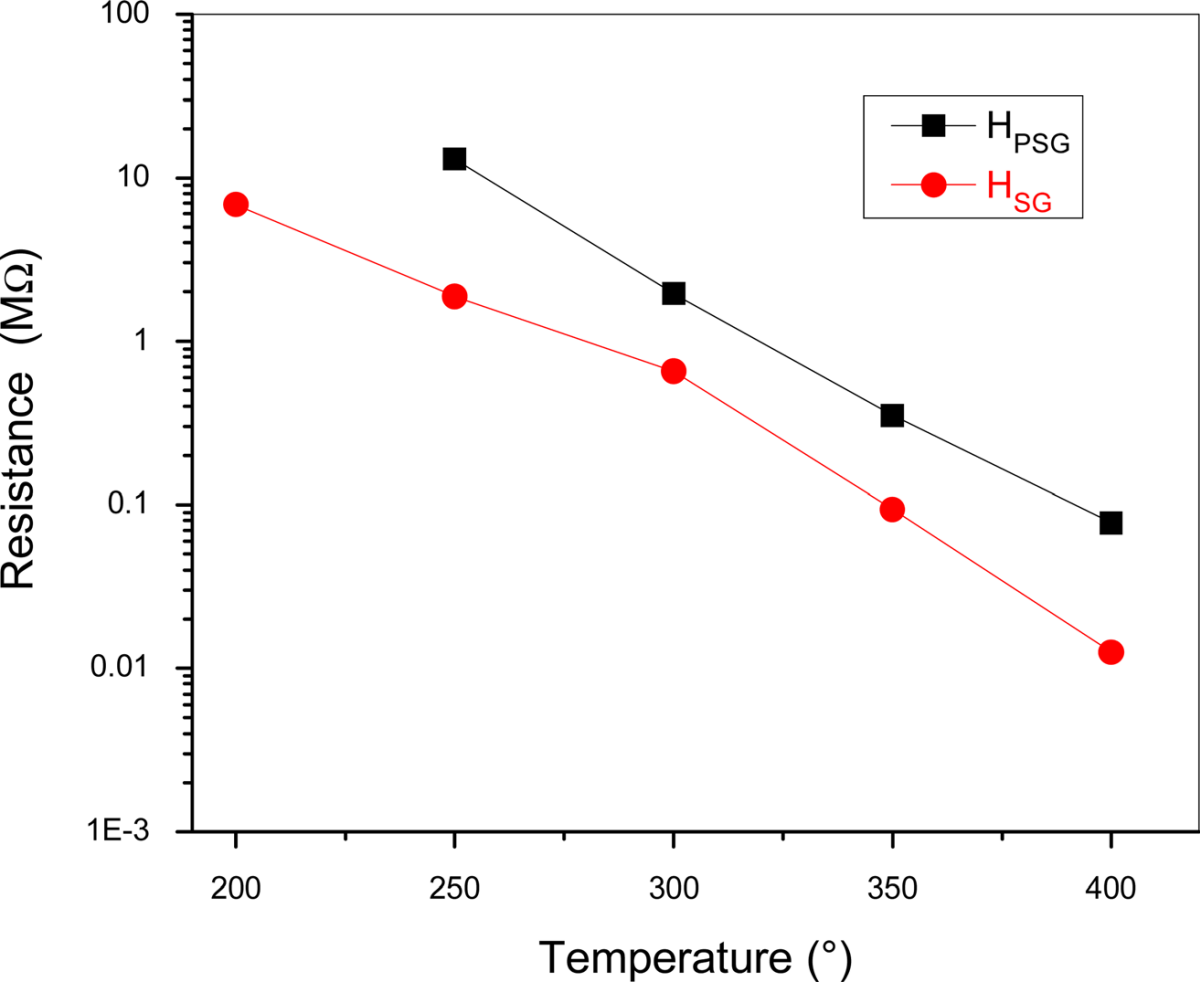
Resistance changes in the samples *versus* temperature in synthetic dry air.

The electrical resistance of the sensors was measured in synthetic dry air by varying the operating temperature from 200 to 400 °C in air (Fig. 4).

At temperature below 200 °C, H_PSG_ sensor resistance cannot be registered by our measurement apparatus due to its high value. Both samples showed a decrease in the resistance with an increase in the temperature. This is an expected behavior since hematite is an n-type semiconductor and the resistance decreased due to the thermal generation and jumping of electrons from the valence band to the conduction band of α-Fe_2_O_3_.^27^ Despite both samples exhibiting the same α-Fe_2_O_3_ phase, the resistance of H_PSG_ was higher than that of H_SG_. The different structural and morphological structure were the main reason of the electrical resistance difference. The higher conductivity of the sample prepared by the sol–gel method was probably attributed to the larger particle size and lesser number of contact points between the particles. Furthermore, it is possible that oxygen vacancies, which act as donor levels, provide free electrons to α-Fe_2_O_3_, leading to higher conductivity.

**Fig. 5:**
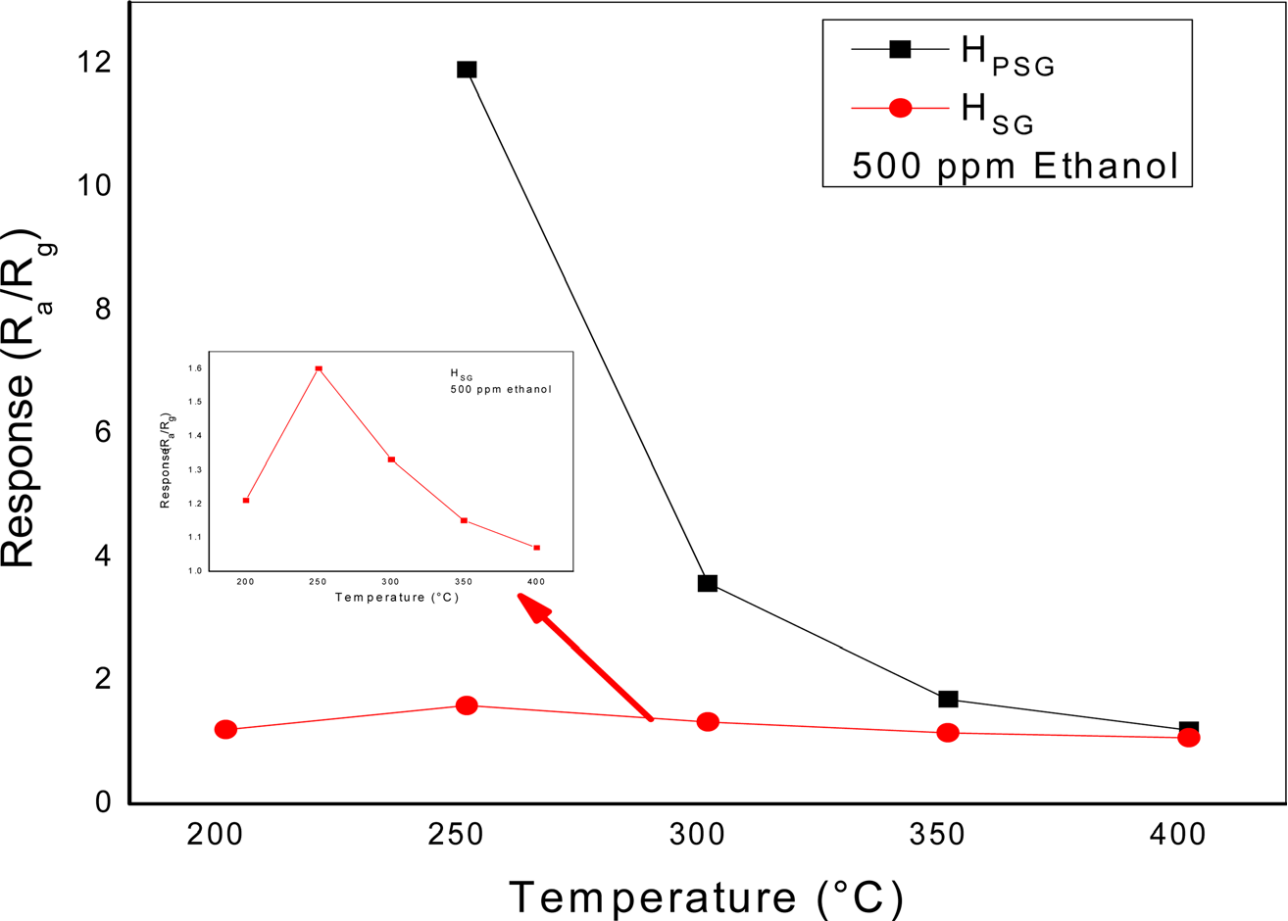
Gas response *versus* the working temperature of the α-Fe_2_O_3_ sensors.

To find the optimum working temperature of the sensors, we exposed the gas response toward 500 ppm ethanol at different temperatures from 200 to 400 °C, as shown in Fig. 5.

For the H_SG_ sensor, the response toward ethanol exhibited a bell shape. The highest response was observed at 250 °C. At lower temperature, the decrease in the sensitivity was probably due to the insufficient activation energy necessary to activate the gas molecules. Also, Dehkordi *et al.* attributed the low response at low temperature to the presence of water molecules on the sensing layer surface.^28^ At temperatures above 250 °C, the response gradually decreased because of the minor concentration of the oxygen species that reacted with ethanol molecules and the higher desorption rate compared with the adsorption rate.^29^ At 250 °C, the response of the H_PSG_ sample was 8 times higher than that of the H_SG_ sample, indicating that the shape and size of the particles strongly affected the sensors' sensitivity. By increasing the operating temperature, the response of both the sensors decreased; hence, further studies were carried out at 250 °C.

**Fig. 6:**
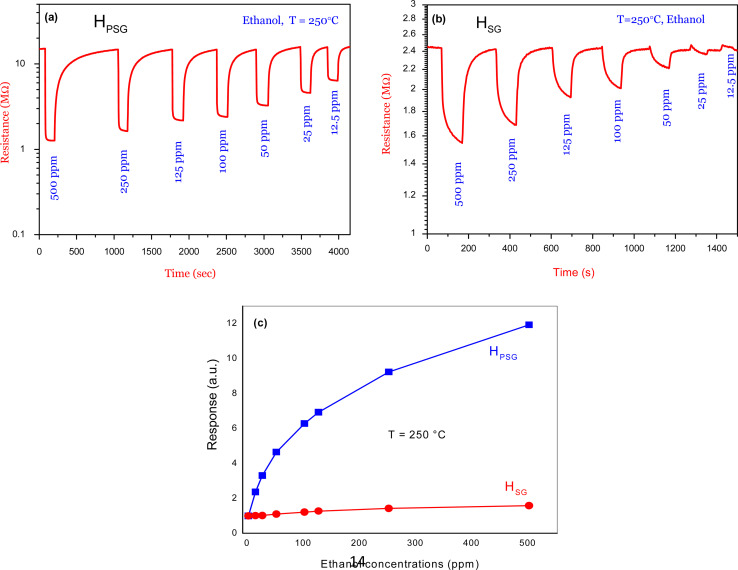
Dynamic resistance curves of (a) H_SG_ sensor and (b) H_PSG_ sensor to various concentrations of ethanol at 250 °C (c) corresponding calibration curves.

Fig. 6(a) shows the transient response of the H_PSG_ sensor toward various ethanol concentrations (12.5 to 500 ppm) at 250 °C. Ethanol exposure led to a large variation in the electrical resistance of the sensing layer. Besides, the signal returned to its initial resistance baseline value in all the pulses, indicating the reversibility of the studied gas adsorption on the surface of the material. A response of about 12 toward 500 ppm of ethanol was observed. In Fig. 6(b), the same behavior was obtained for the H_SG_ sensor toward the same ethanol concentrations but with lower response (1.6 toward 500 ppm) compared to that of H_PSG_. As shown in Fig. 6(c), the H_PSG_ sensor exhibited higher response toward various ethanol concentrations in comparison with the H_SG_ sensor.

Fast response and recovery times are among the performances of a good sensor. A study of these parameters is needed; so, in this context Table 3 shows the response and recovery times for both sensors toward 50 and 500 ppm ethanol at 250 °C.

**Table 3:** Response and recovery times of both gas sensors to 50 and 500 ppm ethanol at 250 °C

Sensors	H_PSG_		H_SG_	
Ethanol concentration	50	500	50	500
Response time (s)	16	5	79	9
Recovery time (s)	190	630	67	112

Both sensors exhibited rapid response time but had a slow recovery time toward 500 ppm ethanol. Also, toward 50 ppm ethanol gas, an increase in response time was observed compared to that at 500 ppm. In contrast, the recovery time seems to be reduced toward 50 ppm ethanol. These behaviors could be explained as follows: toward 500 ppm, several ethanol molecules interacted with adsorbed oxygen species on the sensors' surfaces. This large number of gas molecules forced the adsorption process to happen in a short time and then a rapid response time was obtained. In contrast, the desorption of gas molecules was difficult and took a long time due to the large number of ethanol molecules adsorbed on the sensing layer surface. Thus, long recovery times were observed. On the other hand, toward 50 ppm, less amount of gas molecules interacted with oxygen species; hence, adsorption was slow, while desorption was faster. Thus, we could say that the response time was slow and, in contrast, the recovery time was rapid.

The H_PSG_ sensor shows faster response times toward 50 and 500 ppm, while the H_SG_ sensor presents quicker recovery times toward the two concentrations. Due to the higher surface area of the H_PSG_ sample, more interaction sites are created on the sensor surface in comparison with that on the H_SG_ sample. When ethanol is injected on the sensing layer surface, the gas molecules interact with a large number of oxygen species. Thus, the adsorption process becomes faster, while the ethanol molecules take a large time to be desorbed.

**Fig. 7:**
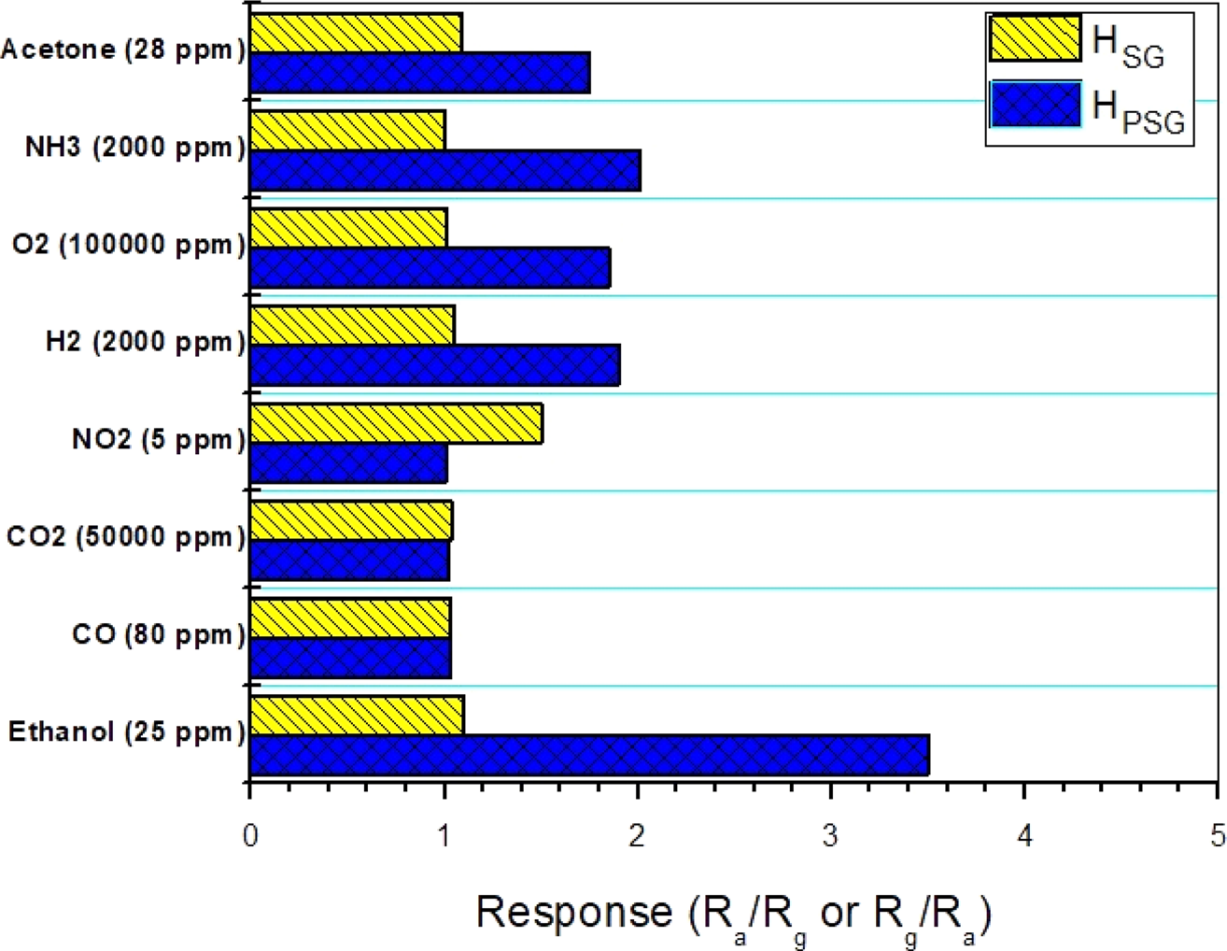
Gas response of the fabricated gas sensors toward several gases at 250 °C.

Selectivity can be defined as the ability to have a high response to a particular gas and less or no response to other gases.^30^ The interfering gases, employed to evaluate the selectivity of the studied sensors, were acetone, ammonia, O_2_, H_2_, NO_2_, CO_2_ and CO. The responses toward different concentrations of interfering gases were measured at 250 °C (Fig. 7). The H_PSG_ sensor showed good selectivity toward ethanol compared to other gases. In fact, at 250 °C, the thermal energy was sufficient to accelerate the reaction between ethanol molecules and the adsorbed oxygen species. On the other hand, other gases needed more thermal energy for interaction.^31^ Thus, the suggested working temperature (250 °C) helped the H_PSG_ sensor to be selective toward ethanol. Besides, the studied gases exhibited various chemical properties. These properties made a difference in the adsorption and catalytic characteristics.^32^ It can be also observed that the H_SG_ sensor had selectivity toward NO_2_ gas as it exhibited a higher response toward this gas among the tested gases. Navale and his team synthesized hematite nanoparticles by the sol–gel technique and found that these nanoparticles had excellent selectivity toward NO_2_ gas.^33^

### 3.3. Ethanol sensing mechanism

Before gas exposure, the adsorbed oxygen species extracted electrons from the hematite surface to form oxygenated anionic species such as O_2_−, O^−^, and O^2−^. Each oxygen species is dominant at a temperature range; in fact, O_2_^−^ appeared at *T* < 100 °C, according to eqn (2), while, O^−^ species was formed at 100 °C < *T* < 300 °C, referring to eqn (3). Finally, O^2−^ was created at *T* > 600 °C using eqn (4).^34,35^

2 O_2_ (ads) + e^−^ ↔ O_2_^−^

3 O_2_^−^ + e^−^ ↔ 2O^−^

4 O^−^ + e^−^ ↔ O_2_^−^

The loss of electrons resulted from the O_2_ molecules adsorption on the n-type α-Fe_2_O_3_ surface, which creates an electronic depletion layer and a high potential energy barrier on the surface of the material, which further leads to an increase in the electrical resistance of the sensor.^36^

After the exposure of the sensor to ethanol gas, a reaction occurred between adsorbed C_2_H_5_OH and O^−^ oxygen species according to eqn (5).^37^

5 C_2_H_5_OH + 8O^−^ → 3CO_2_ + 3H_2_O + 8e^−^

Ethanol, as a reducing gas agent, and the electrons are released back on the sensing layer surface. Since α-Fe_2_O_3_ behaves like an n-type semiconductor, the density of charge carriers was enhanced and, thus, a decrease in the electric resistance was observed in both the samples after gas exposure. After evacuating ethanol from the test chamber, dry air entered, and the oxygen species trapped electrons from the sensing material surface. This led to an increase in the electrical resistance, and then a return to the baseline resistance was achieved.

To explain the gas response enhancement of the H_PSG_ sensor compared to that of the H_SG_ sensor, some factors should be mentioned. XRD analysis showed a difference in the average crystallite size *D* for the two samples; H_PSG_ exhibited the smallest particles size (15 nm). The theoretical surface area (SA) calculated using the formula SA = 6/(*Dρ*),^38^ where *D* is the particles size and *ρ* is the theoretical density of hematite calculated using the Williamson–Hall method mentioned above (5.269 g cm^−3^ for H_PSG_ and 5.255 g cm^−3^ for H_SG_) shows that the SA value is higher for the sample synthesized by the Pechini sol–gel method, *i.e.*, about 57 and 76 m^2^ g^−1^ for the H_SG_ and H_PSG_ samples, respectively. With a larger surface area, more interaction sites are present on the surface of the H_PSG_ material and more electrons are captured on the surface; this led to a large decrease in the resistance after ethanol exposure and higher gas response compared to that of the H_SG_ material. Also, morphological analysis indicated that the H_PSG_ nanoparticles are composed of particles having a peanut-like shape with several open spaces and channels that encouraged ethanol molecules to be diffused in; then, an enhancement in the response was observed. Furthermore, the average sizes of the hematite peanut-like and ellipsoid particles were 0.22 and 1.47 μm, respectively, as shown in Fig. 3(c) and (d). Therefore, in the sensor with lower particles sizes, more contacts were available among particles, which acted as a great source of resistance modulation upon exposure to gases.

In addition to the size, the shape of Fe_2_O_3_ NPs also can affect the sensing performance. However, the main effect of shape of the NPs is the generation of voids among the particles. For the sample prepared using the Pechini sol–gel method, there are many voids and porosities among NPs, facilitating the easy diffusion of the gas into the deep parts of the sensing layer.

## 4 Conclusions

Briefly, α-Fe_2_O_3_ nanoparticles were prepared by two different synthesis methods. Fe_2_O_3_ NPs were synthesized by two sol–gel based synthesis methods. The synthesis procedures were simple and Fe_2_O_3_ with small particle sizes were easily obtained. The advantages of sol–gel processes relative to other synthesis methods are low processing temperature, ease of synthesis, good control over the processing variables, good purity and homogeneity. The sol–gel process gave a sample (H_SG_) composed of particles having an oval shape and average size of about 1.47 μm, while the Pechini sol–gel route (H_PSG_) gave powders containing particles with peanut-like shape and size of 0.22 μm. XRD analysis demonstrated that both the samples were well crystallized. The average crystallite size was 20 nm for the H_SG_ material; it decreased to 15 nm for the H_PSG_ sample. After being tested toward various concentrations of ethanol from 12.5 to 500 ppm, it was clearly shown that the H_PSG_ sensor exhibited the highest response of 12 at 250 °C compared to the H_SG_ sensor. The high response was attributed to the larger surface area and structural characteristics. Although the response time of H_PSG_ (5 s) was faster than that of H_SG_ (9 s), the recovery time was the highest for H_PSG_ (630 s) toward 500 ppm of ethanol. Besides, H_PSG_ presented excellent selectivity toward ethanol when exposed to the gas mixture. All these sensing performances indicated that the H_PSG_ sensor can be a promising candidate for the detection of ethanol.

## Data availability

The data used to support the findings of this study are available from the corresponding author upon request.

## Author contributions

Mokhtar Hjiri: writing and editing; Saja Algessair: data curation; Ramzi Dhahri: conceptualization; Ali Mirzaei: formal analysis; Giovanni Neri: reviewing and supervision.

## Conflicts of interest

The authors declare no conflict of interest.

## Supplementary Material

## References

[cit1] Gao H., Wei D., Lin P., Liu C., Sun P., Shimanoe K., Yamazoe N., Lu G. (2017). Sens. Actuators, B.

[cit2] Hjiri M., El Mir L., Leonardi S. G., Donato N., Neri G. (2013). Nanomaterials.

[cit3] Hjiri M., El Mir L., Leonardi S. G., Pistone A., Mavilia L., Neri G. (2014). Sens. Actuators, B.

[cit4] Jaballah S., Hjiri M., Zahmouli N., Albargi H. B., Dhahri R., Dahman H., El Mir L., Neri G. (2023). J. Mater. Sci.: Mater. Electron..

[cit5] Kong Y., Li Y., Cui X., Su L., Ma D., Lai T., Yao L., Xiao X., Wang Y. (2022). Nano Mater. Sci..

[cit6] Masuda Y. (2022). Sens. Actuators, B.

[cit7] Zhou X., Cao Q., Huang H., Yang P., Hu Y. (2003). Mater. Sci. Eng., B.

[cit8] Li C., Choi P. G., Masuda Y. (2022). Adv. Sci..

[cit9] Li X., Liu C.-X., Zhou J.-R., Ma Y., Ruan S.-P. (2022). Chin. J. Anal. Chem..

[cit10] Tomic M., Fohlerova Z., Gracia I., Figueras E., Cane C., Vallejos S. (2021). Sens. Actuators, B.

[cit11] Singh S., Sharma S. (2022). ACS Omega.

[cit12] Nunes D., Fortunato E., Martins R. (2022). Discover Mater..

[cit13] Wu K., Zhang W., Zheng Z., Debliquy M., Zhang C. (2022). Appl. Surf. Sci..

[cit14] Liang T.-T., Kim D.-S., Yoon J.-W., Yu Y.-T. (2021). Sens. Actuators, B.

[cit15] Jin X., Li Y., Zhang B., Xu X., Sun G., Wang Y. (2021). Sens. Actuators, B.

[cit16] Hjiri M., Aida M. S., Neri G. (2019). Sensors.

[cit17] Balouria V., Kumar A., Samanta S., Singha A., Debnath A. K., Mahajan A., Bedi R. K., Aswal D. K., Gupta S. K. (2013). Sens. Actuators, B.

[cit18] Li D. P., Zhang B. B., Xu J. C., Han Y. B., Jin H. X., Jin D. F., Peng X. L., Ge H. L., Wang X. Q. (2016). Nanotechnology.

[cit19] Umar A., Ibrahim A. A., Kumar R., Albargi H., Alsaiari M. A., Ahmed F. (2021). Sens. Actuators, B.

[cit20] Zhang M., Lu M., Pan H., Bai H., Mei H., Cheng L. (2021). J. Alloys Compd..

[cit21] Li Z., Lin Z., Wang N., Huang Y., Wang J., Liu W., Fu Y., Wang Z. (2016). Mater. Des..

[cit22] Hjiri M., Aida M. S., Neri G. (2019). Sensors.

[cit23] Patil D., Patil L., Amalnerkar D. (2007). Bull. Mater. Sci..

[cit24] Yazirin C., Yazirin C., Puspitasari P., Sasongko M. I. N., Tsamroh D. I., Risdanareni P. (2017). AIP Conf. Proc..

[cit25] Zainuri M. (2017). IOP Conf. Ser.: Mater. Sci. Eng..

[cit26] Williamson G. K., Hall H. (1953). Acta Metall..

[cit27] Srivastava M., Ojha A. K., Chaubey S., Singh J., Sharma P. K., Pandey A. C. (2010). J. Alloys Compd..

[cit28] Dehkordi H. A., Mokhtari A., Soleimanian V., Ghasemi M. (2023). Social Science Research Network.

[cit29] Gong H., Hu J. Q., Wang J. H., Ong C. H., Zhu F. R. (2006). Nano-crystalline Cu-doped ZnO thin film gas sensor for CO. Sens. Actuators, B.

[cit30] Hou S., Pang R., Chang S., Ye L., Xu J., Wang X., Zhang Y., Shang Y., Cao A. (2020). ACS Appl. Mater. Interfaces.

[cit31] Li G., Zhang X., Lu H., Yan C., Chen K., Lu H., Gao J., Yang Z., Zhu G., Wang C., He Z. (2019). Sens. Actuators, B.

[cit32] Xu K., Zhao W., Yu X., Duan S., Zeng W. (2020). Phys. E.

[cit33] Navale S. T., Bandgar D. K., Nalage S. R., Khuspe G. D., Chegoule M. A., Kolekar Y. D., Sen S., Patil V. B. (2013). Ceram. Interfaces.

[cit34] Yamazoe N., Fuchigami J., Kishikawa M., Seiyama T. (1979). Surf. Sci..

[cit35] Skafidas P. D., Vlachos D. S., Avaritsiotis J. N. (1994). Sens. Actuators, B.

[cit36] Chaitongrat B., Chaisitsak S. (2018). J. Nanomater..

[cit37] Qu X., Yang R., Tong F., Zhao Y., Wang M. H. (2018). Powder Technol..

[cit38] Gao L., Li Q., Song Z., Wang J. (2000). Sens. Actuators, B.

